# Endothelium-Derived Hyperpolarizing Factor (EDHF) Mediates Acetylsalicylic Acid (Aspirin) Vasodilation of Pregnant Rat Mesenteric Arteries

**DOI:** 10.3390/ijms221810162

**Published:** 2021-09-21

**Authors:** Helga Helgadóttir, Teresa Tropea, Sveinbjörn Gizurarson, Maurizio Mandalà

**Affiliations:** 1 Department of Biology, Ecology and Earth Sciences, University of Calabria, Arcavacata di Rende, 87036 Cosenza, Italy; helgadottir@hi.is (H.H.); teresa.tropea@manchester.ac.uk (T.T.); 2 Faculty of Pharmaceutical Sciences, University of Iceland, Hofsvallagata 53, 107 Reykjavik, Iceland; sveinbj@hi.is

**Keywords:** endothelial cells, smooth muscle cells, relaxation, pre-eclampsia, hypertension, calcium-activated potassium channels

## Abstract

Acetylsalicylic acid (aspirin) exhibits a broad range of activities, including analgesic, antipyretic, and antiplatelet properties. Recent clinical studies also recommend aspirin prophylaxis in women with a high risk of pre-eclampsia, a major complication of pregnancy characterized by hypertension. We investigated the effect of aspirin on mesenteric resistance arteries and found outdiscovered the molecular mechanism underlying this action. Aspirin (10^−12^–10^−6^ M) was tested on pregnant rat mesenteric resistance arteries by a pressurized arteriography. Aspirin was investigated in the presence of several inhibitors of: (a) nitric oxide synthase (L-NAME 2 × 10^−4^ M); (b) cyclooxygenase (Indomethacin, 10^−5^ M); (c) Ca^2+^-activated K^+^ channels (Kca): small conductance (SKca, Apamin, 10^−7^ M), intermediate conductance (IKca, TRAM34, 10^−5^ M), and big conductance (BKca, paxilline, 10^−5^ M); and (d) endothelial-derived hyperpolarizing factor (high KCl, 80 mM). Aspirin caused a concentration-dependent vasodilation. Aspirin-vasodilation was abolished by removal of endothelium or by high KCl. Furthermore, preincubation with either apamin plus TRAM-34 or paxillin significantly attenuated aspirin vasodilation (*p* < 0.05). For the first time, we showed that aspirin induced endothelium-dependent vasodilation in mesenteric resistance arteries through the endothelial-derived hyperpolarizing factor (EDHF) and calcium-activated potassium channels. By activating this molecular mechanism, aspirin may lower peripheral vascular resistance and be beneficial in pregnancies complicated by hypertension.

## 1. Introduction

The use of aspirin has been growing over the past decades. Initially, it was used as an analgesic, anti-inflammatory, and antipyretic drug, as well as an antiplatelet agent due to its ability to inhibit platelet aggregation [1,2]. Recently, aspirin has been suggested for use in pregnancy with high cardiovascular risk to prevent the development of gestational hypertensive disorders, such as pre-eclampsia (PE) [3,4]. PE is a major complication of pregnancy and is one of the leading causes of maternal and perinatal morbidity and mortality [5]. Currently, there is no cure for PE; the most effective management of the disease is delivery [6], which can worsen neonatal outcomes if it needs to be initiated early in pregnancy. Therefore, early-onset PE requires treatments that prevent preterm birth to enable optimal intrauterine fetal growth [6].

Clinical studies investigating the benefits of administering aspirin in PE produce contradictory results. In all likelihood, it is due to the different gestation period that aspirin treatment was initiated and also to the different doses used. In fact, the benefit of aspirin was only observed in clinical studies that initiated aspirin treatment before the 16th week of pregnancy and at low- and mid-dose [7]; in contrast, aspirin in late pregnancy and at a high dose did not show beneficial effect [8]. However, the mechanisms through which aspirin acts remain unclear. Few studies have suggested a beneficial effect of aspirin on endothelial function, vascular activity, and a possible overall positive effect on the vascular system [9,10,11,12,13,14,15].

In normal pregnancy, the maternal vasculature undergoes a significant change [16] to meet the needs of the placental–fetal growth, including a reduction in the total peripheral vascular resistance (PVR) [17]. On the other hand, PE is characterized by an increase in PVR as a consequence of endothelial dysfunction [18]. The endothelium, the inner layer of the blood vessels, plays an important role in the regulation of the vascular tone by the release of several relaxing and contracting factors that act on the nearby smooth muscle cells [19,20]. In PE, an imbalance between the endothelial-derived factors, named endothelial dysfunction, has been reported [21], associated with microcirculatory disorder [22,23].

Vascular studies of PE are often carried out on the mesenteric vasculature, since it contributes significantly to the total PVR in pregnancy. In this study, we used mesenteric resistance arteries (MAs) from pregnant rats to investigate the effect and the mechanism of action of aspirin on the systemic vasculature. Our results show, for the first time, the molecular mechanisms that underlie aspirin vasodilation of mesenteric arteries, and suggest the splanchnic circulation as a possible target and aspirin benefit in hypertensive disorders of pregnancy.

## 2. Results

**Figure 1:**
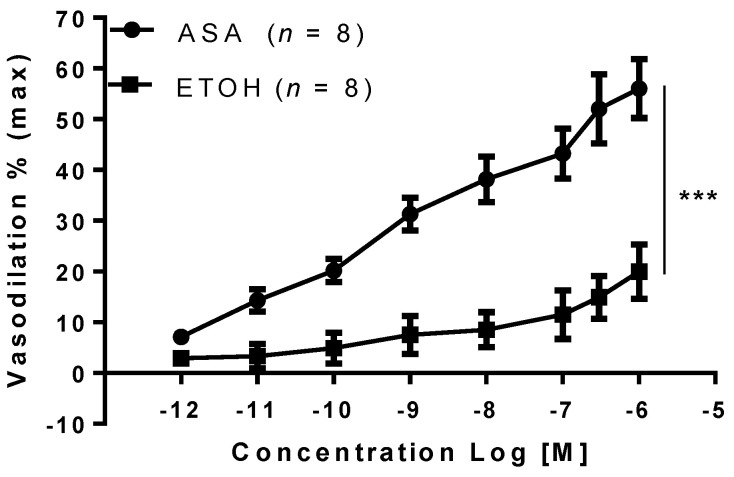
Acetylsalicylic acid vasodilation on mesenteric arteries. Acetylsalicylic acid (ASA) and its vehicle ethanol (ETOH) were tested on phenylephrine preconstricted mesenteric arteries isolated from gravid rats. Data are reported as mean ± SEM, *n* = number of experiments, *** *p* < 0.001 (two-way ANOVA).

Aspirin was tested on preconstricted MA and induced a dose-dependent vasodilation, reaching 62 ± 11% at the highest concentration of 10^−6^ M. Meanwhile, the vehicle for aspirin, ethanol, induced much less vasodilation (22 ± 6%, at 10^−6^ M), which was statistically different compared to the vasodilation caused by aspirin (*p* < 0.001, Figure 1).

**Figure 2:**
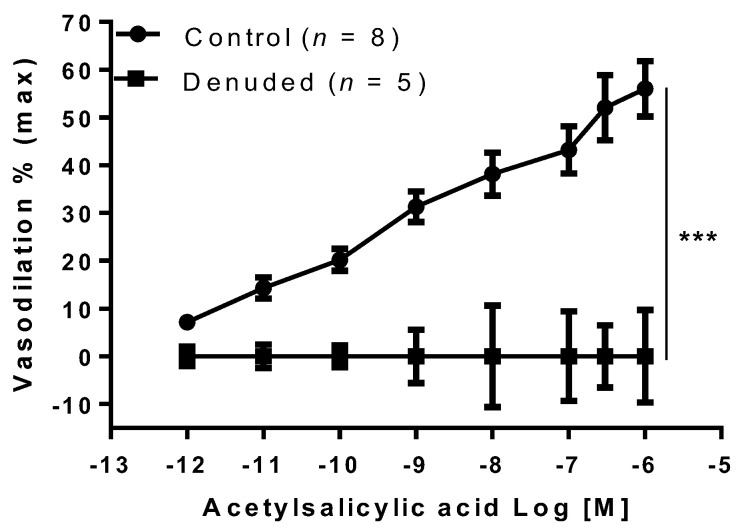
Acetylsalicylic acid effect on entire and denuded mesenteric arteries. Acetylsalicylic acid was tested on phenylephrine preconstricted mesenteric arteries entire (Control) and without endothelium (Denuded) isolated from gravid rats. Data are reported as mean ± SEM, *n* = number of experiments. *** *p* < 0.001 (two-way ANOVA).

To determine whether the endothelium was involved in the aspirin vasodilation, a dose–response to aspirin was carried out on both intact and denuded MA. Interestingly, removal of the endothelium completely abolished the vasodilation in response to aspirin (Figure 2).

**Figure 3:**
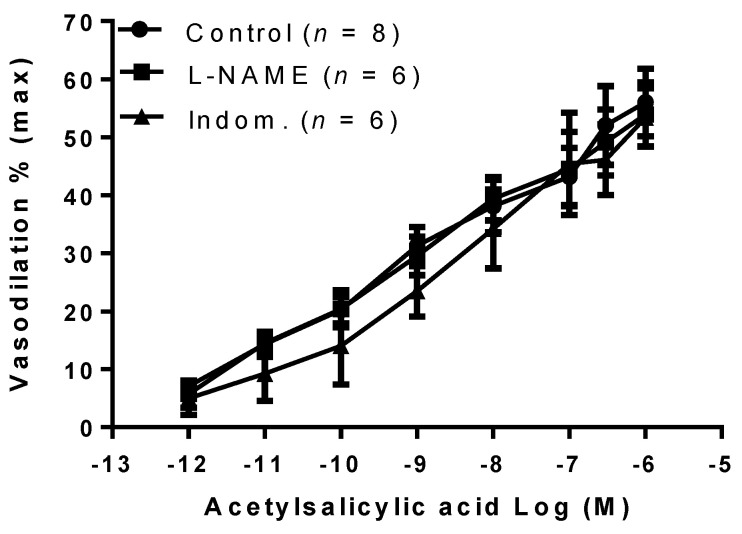
Effect of acetylsalicylic acid in the presence of nitric oxide synthase and cyclooxygenase inhibitors. Acetylsalicylic acid was tested on phenylephrine preconstricted mesenteric arteries isolated from gravid rats in the absence (Control) and in the presence of inhibitors of either nitric oxide synthase (L-NAME, 2 × 10^−4^ M), or cyclooxygenase (Indom., 10^−5^ M). Data are reported as mean ± SEM, *n* = number of experiments.

Then, to determine the endothelial-derived relaxation factors involved in the vasodilation of aspirin, it was tested in the presence of inhibitors of NOS (L-NAME, 2 × 10^−4^ M) and COX (indomethacin, 10^−5^ M). Vasodilation to aspirin was not affected by either L-NAME or indomethacin (Figure 3).

**Figure 4:**
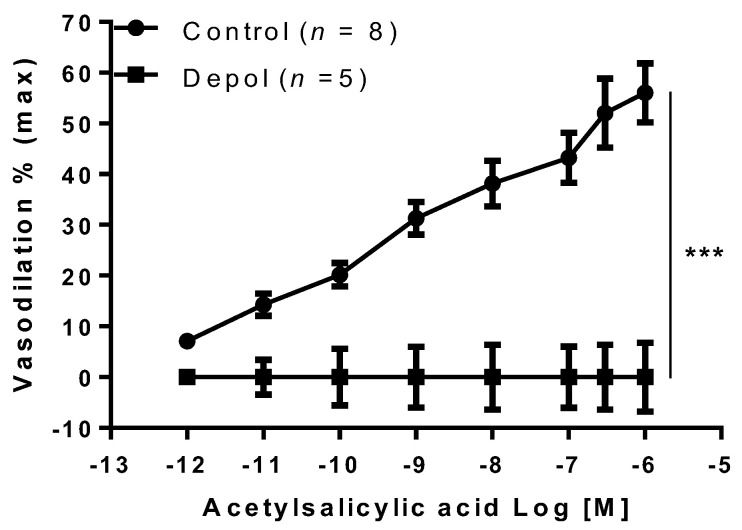
Effect of acetylsalicylic acid on preconstricted arteries. Acetylsalicylic acid was tested on mesenteric arteries isolated from gravid rats, preconstricted with phenylephrine (Control) or with high KCl (80 mM), which induces depolarization (Depol). Data are reported as mean ± SEM, *n* = number of experiments, *** *p* < 0.001 (two-way ANOVA).

To find out if the EDHF was involved in aspirin vasodilation, MA were preconstricted with high KCl (80mM), which completely abolished the vasodilator effect of aspirin (*p* < 0.001, Figure 4).

**Figure 5:**
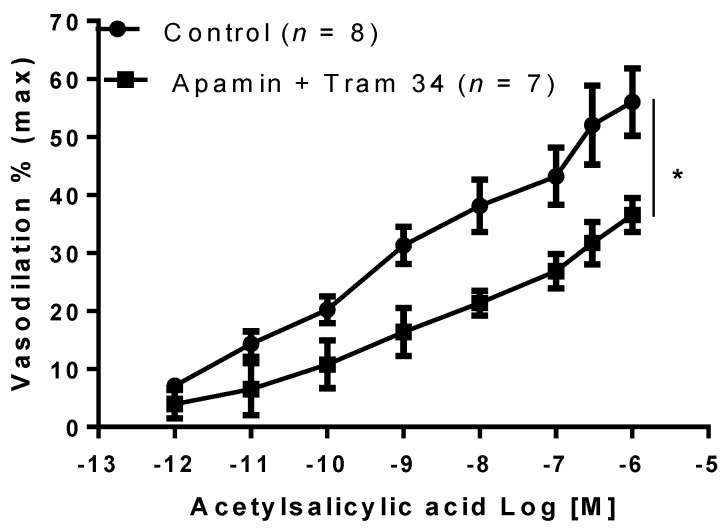
Effect of acetylsalicylic acid in the presence of calcium-activated potassium channel inhibitors. Acetylsalicylic acid was tested on phenylephrine preconstricted mesenteric arteries isolated from gravid rats in the absence (Control) or in the presence of both calcium-activated potassium channel small (Sk_Ca_,) and intermediate (IK_Ca_) inhibitors Apamin (10^−7^ M) and TRAM-34 (10^−5^ M), respectively. Data are reported as mean ± SEM, *n* = number of experiments, * *p* < 0.05 (two-way ANOVA).

In addition, vasodilation to aspirin was significantly reduced following pre-incubation with apamin (10^−7^ M, *p* < 0.05) and TRAM-34 (10^−5^ M, *p* < 0.05) blockers of SKca and IKca channels, respectively (Figure 5).

**Figure 6:**
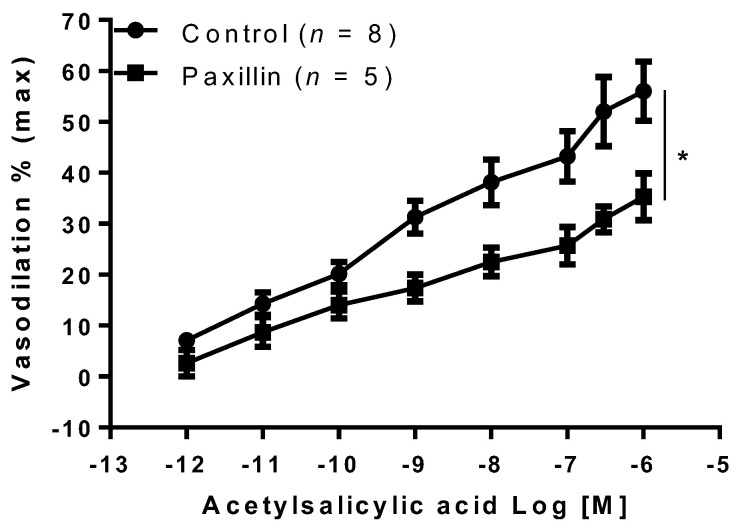
Effect of acetylsalicylic acid in the presence of BKca channel inhibitor. Acetylsalicylic acid was tested on mesenteric arteries isolated from gravid rats in the absence (Control) or in the presence of big potassium channel (BKca) inhibitor (paxillin, 10^−5^ M). Data are reported as mean ± SEM, *n* = number of experiments, * *p* < 0.05 (two-way ANOVA).

Furthermore, additional experiments demonstrated that vasodilation was significantly reduced in MA preincubated with the BKca channel inhibitor, paxillin (10^−5^ M, *p* < 0.05, Figure 6).

## 3. Discussion

This study demonstrates that aspirin is a potent vasodilator of resistance MA isolated from pregnant rats, and it acts in a dose-dependent manner. The maximum vasodilation of about 62% was achieved at 10^−6^ M, although the relaxation effect was already observed at a very low concentration of 10^−11^ M, suggesting a high sensitivity of MA to aspirin.

Our results demonstrated that aspirin vasodilation of MA is endothelium-dependent, is mediated by the EDHF, and involves the calcium-activated potassium channels, SKca, IKca, and BKca.

Our data on aspirin vasodilation effect agree with previous studies in small uterine arteries (UA) [13], as well as in rat aortic rings [11], suggesting that aspirin acts on both resistance and conduit arteries. Small resistance arteries play an important role in the regulation of the blood flow to the organs, contributing to the total PVR, whose changes strongly influence blood pressure [24].

We carried out the experiments on resistance MA, which contributes significantly to total PVR since the mesenteric circulation receives approximately one third of cardiac output [25].

Our results demonstrate that aspirin vasodilation of MA is completely endothelium-dependent, since denuded MA were unresponsive to aspirin. This finding is in conformity with a previous work on UA [13]. However, in UA, aspirin vasodilation was mediated by nitric oxide (NO) and prostacyclin (PGI2) pathways, while, in MA, it occurred via activation of EDHF. These differences in the two types of vascular beds, one reproductive and the other systemic, suggest a vascular region dependency for the mechanisms underlying the aspirin vasodilation. Our results highlight the important role of EDHF in the aspirin vasodilation of resistance MA, in agreement with studies showing that EDHF is the major mediator of dilatation in the mesenteric vascular bed from pregnant rats [26], while, in UA, vasodilation is mainly mediated by NO [27].

Interestingly, EDHF vasodilation is reduced in complicated pregnancy, such as PE [28], with consequent increases in PVR, a hallmark of hypertension [29,30,31,32,33]. Therefore, on the basis of our results, we speculate that aspirin may benefit pregnancy with hypertension through lowering peripheral vascular tone by activating EDHF.

Several candidate molecules have been proposed for EDHF, such as cytochrome-P450-derived arachidonic acid metabolite [34] myoendothelial gap junctions [35] and K^+^ channels [36]. In microcirculatory vascular beds, it is generally accepted that endothelial SK_ca_ and IK_ca_ play a pivotal role in mediating EDHF effects [37].

Our results showed that aspirin effect was reduced by the inhibition of SK_ca_ and IK_ca_, suggesting that the activation of those channels is involved in aspirin vasodilation of MA. Although, in the present study, the contribution of each channel was not studied, this possibility requires further investigation.

In addition, we also showed the involvement of BKca channels in the aspirin vasodilation, since it was reduced by the inhibitor paxillin. This can be interpreted by the support of the EDHF being the molecule that activates smooth muscle cell BKca channels, leading to hyperpolarization and vasodilation.

The Kca channels have been reported to be involved in the hyperpolarization and vasodilation of mesenteric arteries [38,39]. This is in agreement with our results, showing that aspirin vasodilation was completely inhibited by depolarization induced by high potassium chloride.

## 4. Materials and Methods

### 4.1. Animals

All experiments were conducted in accordance with the ‘3R principles’ (www.nc3rs.org.uk, accessed on 24 February 2017) to reduce the number of animals and to optimize experimental protocols for obtaining maximum data from each tested animal, and with the European Guidelines for the care and use of laboratory animals (Directive 2010/63/EU). The arteries were isolated from animals used in a study approved by the local ethical committee at the University of Calabria and the Italian Ministry of Health (n.74/2018-PR).

Sprague–Dawley 12–14-week-old gravid rats at term (20 days of gestation) were used in the experiments. Animals were housed at a temperature-controlled condition of 22 °C ± 2 °C and under a 12-h light/dark cycle; food and water were provided ad libitum. Females were bred overnight in isolated pairs by the placement of a male rat. If a seminal plug was observed the following morning, the first day of pregnancy was confirmed.

The animals were first euthanized with isoflurane (4%) and then decapitated; once dead, the abdominal cavity was opened and the entire mesentery was dissected and placed in a Petri dish containing cold (4 °C) HEPES physiological salt solution (HEPES-PSS).

### 4.2. Vessel Preparation

Arterial segments (2–3 mm) of mesenteric resistance arteries (MA, diameter <300 µm) were obtained from rats. The segments were dissected free from connective and adipose tissue and transferred to a chamber of a small-vessel arteriography (Living Systems Instrumentation, St. Albans City, VT, USA). One end of the vessel was tied onto a glass cannula and flushed of any luminal content by increasing the pressure before securing the distal end onto a second cannula using a servo-null pressure system (Living Systems Instrumentation, St. Albans City, VT, USA). Before using the vessels for the experiments, the functionality of both smooth muscle and endothelial cells was tested, respectively, by high KCl (80 mM) and acetylcholine (10^−5^ M). The vessels that did not respond to both compounds were discarded.

### 4.3. Experimental Protocol

All vessels were continuously superfused with HEPES-buffered physiological saline solution (HEPES-PSS) at 37 °C and pH 7.4. Each vessel was pressurized to 50 mmHg (value that imitates in vivo conditions) and equilibrated for 45–60 min before the beginning of each experimentation. Lumen diameter was measured by transilluminating each vessel segment using a video dimension analyzer (Living Systems Instrumentation, St. Albans City, VT, USA) in conjunction with data acquisition software (Ionoptix, Westwood, MA, USA) to continuously record lumen diameter. Following equilibration, all vessels were preconstricted with phenylephrine to produce a 40–50% reduction in baseline diameter [40]. Once constriction was achieved and stable for about 10 min, aspirin was added in the range of concentration of 10^−12^ to 10^−6^ M, and the resulting vasodilation was recorded. Moreover, ethanol (ETOH), as a vehicle of aspirin, was tested on phenylephrine-preconstricted artery at the same amount present in the range 10^−12^–10^−6^ M of aspirin.

Aspirin was tested in both intact and denuded (without endothelium) arteries. The endothelium was removed by air perfusion and the effectiveness of this procedure was confirmed by the absence of endothelium-dependent vasodilatation to acetylcholine (10^–5^ M).

To investigate the mechanism of action of aspirin, additional experiments were performed on intact arteries using: (a) Nω-nitro-L-arginine methyl ester (L-NAME, 2 × 10^−4^ M) to block nitric oxide synthase (NOS); (b) indomethacin (10^−5^ M) to block cyclooxygenase (COX); (c) paxillin (10^−5^ M) to block BK_Ca_ channels; (d) apamin (10^−7^ M) to block Sk_Ca_ channels; (e) TRAM-34 (10^−5^ M) to block IK_Ca_ channels; and (f) HEPES-PSS high KCl (80 mM) to block endothelial-derived hyperpolarizing factor (EDHF) vasodilation. Vessels were preincubated with the inhibitors for 20 min before preconstriction with phenylephrine, and then aspirin was tested.

Aspirin vasodilation was expressed as percent of maximal diameter, which was determined at the end of each experiment in the presence of a relaxing HEPES-PSS solution containing diltiazem (10 µM) and papaverine (100 µM).

### 4.4. Materials

The physiological salt solution HEPES-PSS was freshly prepared for each experiment and comprised of: NaCl (141.8 mM), KCl (4.7 mM), MgSO_4_ (1.7 mM), EDTA (0.5 mM), CaCl_2_ (2.8 mM), HEPES (10.0 mM), KH_2_PO_4_ (1.2 mM), and glucose (5.0 mM). The pH was adjusted to 7.4 at 37 °C with 10 M NaOH. In HEPES-PSS high KCl (80 mM), the composition was the same as HEPES-PSS, except for equimolar substitution of KCl for NaCl.

Chemicals: phenylephrine, L-NAME, indomethacin, paxillin, apamin and all the above compounds for the HEPES-PSS preparationwere purchased from Sigma-Aldrich (Milan, Italy), while TRAM-34 was purchased from Abcam (Cambridge, UK). Acetylsalicylic acid (aspirin, Rhodine^®^ 3118) was kindly provided by Novacyl (Lyon, France).

### 4.5. Statistics

Data were expressed as mean ± SEM, where *n* is both the number of arterial segments studied and animal used. Data were analyzed for normal distribution by Shapiro–Wilk test. Differences in responses between groups were determined by two-way ANOVA, as indicated in figure legends. *p* values ≤ 0.05 were considered statistically significant.

## 5. Conclusions

Our results provide an insight into the molecular mechanism underlying aspirin vasodilation of resistance MA, and suggest that aspirin may be beneficial for the pregnant cardiovascular system by lowering PVR and, therefore, blood pressure.
